# West Nile Virus Lineage 2 Neuroinvasive Infection Presenting as Intraparenchimal Cerebral Hemorrage

**DOI:** 10.3390/healthcare14050607

**Published:** 2026-02-27

**Authors:** Antonio Mastroianni, Simone Malagò, Valeria Vangeli, Giuliana Guadagnino, Luciana Chidichimo, Maria Vittoria Mauro, Francesca Greco, Robert Tenuta, Lavinia Berardelli, Antonio Mori, Sonia Greco, Concetta Castilletti

**Affiliations:** 1 Infectious and Tropical Diseases Unit, “Annunziata” Hub Hospital, Azienda Ospedaliera di Cosenza, Viale della Repubblica s.n.c., 87100 Cosenza, Italy; valeriavangeli@gmail.com (V.V.); giuliana.guadagnino@gmail.com (G.G.); chidichimoluciana@gmail.com (L.C.); lavinia.berardelli@gmail.com (L.B.); grecosonia1976@gmail.com (S.G.); 2 Virology and Emerging Pathogens Unit, Department of Infectious—Tropical Diseases and Microbiology, IRCCS Sacro Cuore Don Calabria Hospital, 37024 Negrar di Valpolicella, Italy; simone.malago@sacrocuore.it (S.M.); antonio.mori@sacrocuore.it (A.M.); concetta.castilletti@sacrocuore.it (C.C.); 3 Microbiology Unit, “Annunziata” Hub Hospital, Azienda Ospedaliera di Cosenza, 87100 Cosenza, Italy; m.v.mauro@virgilio.it (M.V.M.); francesca.greco@aocs.it (F.G.); r.tenuta@aocs.it (R.T.)

**Keywords:** West Nile virus, stroke, cerebral vasculitis, cerebral hemorrage, Guillain-Barré syndrome, facial palsy, arbovirus

## Abstract

**Objective**: The aim of this retrospective study was to evaluate clinical and laboratory characteristics in adult patients with neuroinvasive West Nile virus (WNDD). We also studied the phylogeny and molecular characteristics of some of the WNV strains. **Methods**: A retrospective analysis was conducted at “Annunziata” Hub Hospital, a secondary referral facility in Calabria region, in Southern Italy. Sample pre-processing, sequencing and bioinformatic analyses were carried out at IRCCS Sacro Cuore Don Calabria Hospital in Negrar di Valpolicella, Verona, Veneto region in North-East Italy. **Results**: Nine cases of WNDD were analyzed, involving eight males and one female, with a mean age of 70.33 years (range 60–85). The overall average hospital stay was 20.6 days (range 6–46). Six patients made a full recovery after a mean of 35.3 days of acute care. Thirty-day mortality rate was 23%. VNDD in some of our patients manifested itself in the form of cerebral hemorrhage (ICH) in three patients, causing lethality in two patients and other unusual manifestations, such as Guillain–Barré syndrome with fatal outcome and severe facial palsy. Phylogenetic analysis shows that our sequences are closely related to other southern-Italian and cluster with Central–Southern–Eastern European sequences, while being evidently separated from northern Italian and Central–Western European ones, belonging to the sub-lineage 2a of the WNV-2, clustering with sequences from the Central–South–Eastern clade, mainly to Hungary. **Conclusions**: Cerebrovascular complications of WNE may be an important clinical manifestation of WNV neuroinvasive infection. Preliminary data do not allow us to determine whether our strains, closely related to other southern-Italian and cluster with Central–Southern–Eastern European sequences, really presented an increased neurovirulence.

## 1. Introduction

Most West Nile Virus (WNV) infections are asymptomatic, mild, or undiagnosed; however, severe cases of WNV neuroinvasive disease (WNND) are incresingly reported, probably related to WNV’s specific affinity for the nervous system, both central and peripheral. West Nile encephalitis (WNE) presents as severe neuroinvasive disease after a flu-like prodrome, featuring rapid progression to neurological issues, like acute flaccid paralysis, movement disorders, altered mental status, seizures, and neck stiffness, affecting under 1% of WNV-infected individuals [1].

WNE involves brain inflammation, but it can also trigger cerebrovascular issues like vasculitis and strokes, both ischemic and/or hemorrhagic, making it mimic a stroke or cause stroke-like symptoms. This neuroinvasive form (<1% of cases) damages blood vessels, leading to these serious complications, even causing intracranial cerebral hemorrage (ICH), expanding the range of severe WNV neurological impacts beyond typical clinical features [2].

ICH is a leading cause of death worldwide and an important health issue. Although common causes predominantly affect the elderly, there exists a spectrum of uncommon etiologies that contribute to the overall incidence of ICH [3].

Infectious causes of ICH may involve organisms like bacteria (Streptococcus pneumoniae, Treponema pallidum), viruses (HIV, VZV, HSV, COVID-19), fungi (Aspergillus, Cryptococcus), and parasites, leading to inflammation (vasculitis), ruptured aneurysms, or clotting issues (coagulopathy) that damage blood vessels, causing them to leak or burst, as seen in tuberculous meningitis, endocarditis, or invasive fungal infections [3].

During the summer of 2025, we consecutively followed two patients with WNE complicated by ICE (Table 1, patient 7 and 9), among other patients. This occurrence raised our clinical suspicion of a potential correlation between WNE and the risk of ICH, also considering a case of WNE complicated by ICH observed in 2024 (Table 1, patient 2). For this reason, we decided to reevaluate the clinical features of the WNE cases observed in our department and compare our results with an extensive literature review.

## 2. Methods

We conducted a descriptive study of cases of WNDD occurred between 2023 and 2025 in the province of Cosenza, which covers a large territory in Calabria, a region of Southern Italy, in order to evaluate unusual clinical presentations. Nine cases of WNND were analyzed, involving 8 males and 1 female, with a mean age of 70.33 years (range 60–85) (Table 1). WNE was confirmed by the demonstration of specific IgM and IgG antibodies in serum using routine serological screening tests.

Laboratory diagnosis of WNDD was carried out at the “Annunziata” Hub Hospital, Cosenza. Cerebrospinal fluid (CSF) and blood sample were analyzed for WNV infection using the one-step real time RT-PCR (kit WNV ELITe MGB® Kit (ELITechGroup SAS, Puteaux, France), following the manufacturer’s instructions. All patients had specific IgM antibodies at admission and IgG antibodies were present in 90%.

We investigated the phylogeny and molecular characteristics on the complete genome sequence of the GBS case, identified in September 2023 (WNV-2-Cal/2023), as well as the complete genome sequences of two of the patient with ICH ascociated WNE in August 2024.

The analysis included sequences from the GenBank database, ranging from the first European/African sequences deposited up to September 2025. The majority of sequences originate from Italy (*n* = 124), Greece (*n* = 112), Germany (*n* = 99), Russia (*n* = 87), and Hungary (*n* = 76), primarily associated with mosquito (~36%), humans (~29%), and birds (~27%) hosts.

Sample pre-processing, sequencing and bioinformatic analyses were carried out at IRCCS Sacro Cuore Don Calabria hospital in Negrar di Valpolicella, Verona. All the analyses were performed on the same whole blood samples used for the laboratory diagnosis.

The whole blood samples’ nucleic acid content was extracted via Qiagen EZ1 Advanced XL, with the EZ1 ^®^ DSP Virus kit, from 200 uL into 60 uL (Qiagen, Hilden, Germany). RNA was quantified using the Qubit RNA HS assay kit (Invitrogen, Thermo Fisher Scientific, Inc., Waltham, MA, USA) and the High Sensitivity RNA ScreenTape on the 4200 TapeStation System (Agilent Technologies Inc., Santa Clara, CA, USA).

Full-length WNV genome sequences were obtained using two different approaches: (i) the Illumina RNA prep kit with enrichment, with Viral Surveillance Panel (VSP) hybridization capture probes (Illumina, San Diego, CA, USA), following the manufacturer’s instructions; and (ii) a panel of tiled amplicons of 400 bp in length. Amplicons underwent Illumina DNA prep kit processing, as per manufacturer instructions. Libraries were loaded on Illumina P1 flow cells (Illumina, San Diego, CA, USA), running 2 × 150 bp, on a NextSeq1k. Each library was evaluated via qubit DNA HS and High Sensitivity DNA ScreenTape 5000 prior to loading on the appropriate flow cell.

## 3. Results

The overall average hospital stay was 20.6 days (range, 6–46). Six patients made a full recovery with no neurological sequelae after a mean of 35.3 days of acute care. Thirty-day mortality rate was 23%.

Three male patients with a median age of 71 years (range 68–74), presented ICH associated with WNE (Table 1, patient 2, 7, 9). Two male patients, the first 74-year-old (Table 1, patient 2) suffering from arterial hypertension and the second 68-year-old (Table 1, patient 9) suffering from diabetes mellitus and post-ischemic dilated heart disease, died after 13 and 21 days, while the third patient (Table 1, patient 7) was transferred to a neuromotor rehabilitation facility after 34 days of hospitalization. This patient, before admission to the hospital, presented age-related brain involution symptoms and suffered from chronic hypertensive heart disease. The reasons for admission to the emergency room were worsening motor difficulties with weakness in the lower limbs, a rapidly worsening state of confusion, high fever, and an episode of hypertensive peak. Repeated studies of the brain and spinal cord, using computed tomography (CT) scans and magnetic resonance imaging (MRI), have documented a very complex picture, characterized by diffuse meningoencephalitis, with a diffuse flogistic involvement of the pachy-leptomeninges and the equine cauda roots, blood deposits in the occipitopolar and bihemispheric mid-posterior cingulate sulci, the left temporoparietal carrefour, the fourth ventricle and occipital horns and the pericerebellar cistern. There was also evidence of a diffuse posterior dorso-lumbo-sacral, anterior dorso-lumbar (up to L1) and sacral spinal cord epidural hematoma. Diffuse hemosiderin coating was present on the dural surface in the dorso-distal posterior lumbo-sacral and anterior lumbo-sacral regions, as well as at the anterior 8th-10th level. Serology for WNV was positive, while WNV RNA testing in CSF, blood, and urine was negative. Treatment included two 5-day cycles of high-dose immunoglobulins and the use of dexamethasone (Table 1, patient 7).

The second patient was a 68-year-old man with diabetes mellitus and post-ischemic dilated heart disease, admitted to the emergency room for a syncope in absence of cardiac arrhythmias (Table 1, patient 9).

The patient appeared drowsy, but initially arousable. A spinal tap was not performed urgently due to concomitant aspirin therapy. A brain CT scan documented an increase in the volume of the subarachnoid spaces of the vault and skull base, thin, hygromatous subdural hypodense layers in the bilateral frontal lobe, and a blood hyperdensity within the right subdural collection in the right frontotemporal parietal lobe. An EEG revealed slow background activity in the theta and delta bands, with low voltage, and slightly asymmetric (right greater than left). Serology for WNV was positive and urine WNV-RNA testing was positive with a titer of 1072 copies/mL The patient received both high-dose immunoglobulin for seven days and dexamethasone, but his clinical condition did not change, resulting in death 13 days after hospital admission.

The third patient was a 74-year-old male (Table 1, patient 2) suffering from hypertensive heart disease, hospitalized for high fever, worsening spatial-temporal disorientation and mental confusion, associated with dysarthria. There was evidence of a left upper limb paresis. An EEG revealed a widespread slowing of brain bioelectrical activity to 4–5 c/s, interspersed with additional bursts of spike waves over the left frontotemporal regions, which spread to the contralateral hemisphere. Both a CT scan and a brain MRI documented subdural hygromatous collections along the bilateral fronto-temporo-parietal convexities. Despite treatment with high-dose immunoglobulin for 5 days and dexamethasone, the clinical conditions worsened, resulting in death 21 days after hospital admission. Four patients (Table 1, patient 3, 4, 5, 6) presented with moderate severity of WNE, responded positively to treatment with immunoglobulins and dexamethasone and were discharged with complete recovery.

In our small cohort, we observed two other cases of patients with an unusual presentation. A 66-year-old male patient presented with severe right Bell’s palsy, which gradually regressed over approximately two months. This patient was treated with both steroids and immunoglobulins, leaving no neurological deficits and without relapse at the six-month follow-up. Steroid treatment was continued with tapering doses for approximately six weeks. This is the first patient with WNV-related facial palsy to be treated simultaneously with steroids and immunoglobulins, with a complete clinical response.

A 63-year-old man (Table 1, patient 5) developed a fulminant Guillain–Barré syndrome (GBS) responsible for a severe tetraparesis and a respiratory failure, requiring a tracheostomy and intubation. He was transferred to a rehabilitation long-term care facility 46 days after hospital admission, but he died after approximately two months, because of severe respiratory complications.

**Figure 1:**
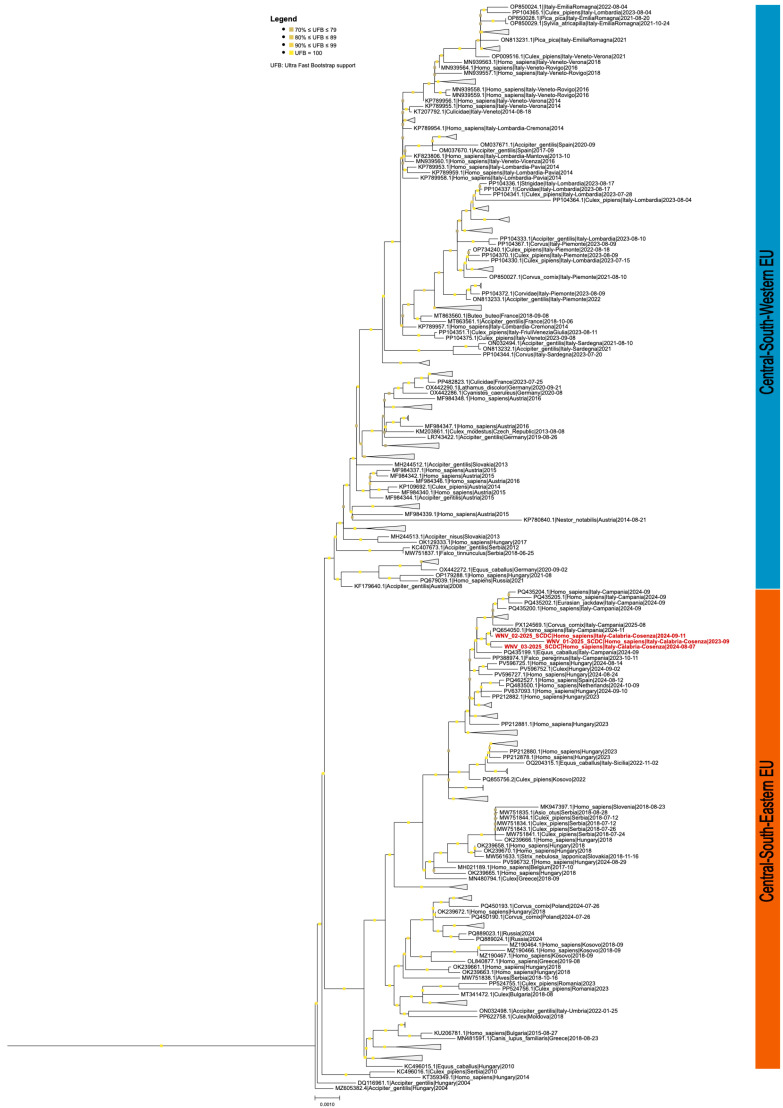
Phylogenetic analysis of WNV lineage 2 whole-genome sequences. In this sub-selection only European sequences were included. Red-colored labels refer to clinical cases collected at out hospital. Colored bars on the right delineate the separation between Central–Western European (blue) and Central–Southern–Eastern (red) sequences. Bootstrap values are reported where ≥90. The original tree comprises European and African sequences from the first deposited whole-genome sequence up to August 2025. For visualization constraint, clades that contain either multiple records of the same country or low bootstrap values were collapsed. To inspect the full phylogenetic tree see microreact.

Phylogenetic analysis showed that the WNVIRCCS-SCDC_01/2025 strain (WNV2-Cal/2023) and the other two strains analyzed belonged to the sub-lineage 2a of the WNV-2, clustering with sequences from the Central–South–Eastern clade (Figure 1). The neighboring branches reported sequences from the Southern and Eastern Europe, such as Greece, Hungary, Serbia, Russia, Romania, Slovakia, Poland and Kosovo. In particular, the most closely related sequences could be traced back mainly the WNV Hungary 578/10 strain (GenBank accession ID KC496015.1). The polyprotein sequence analysis of the WNV-2 strains from Calabria region identified non-synonymous substitutions that were representative signatures in the genome-based phylogenetic analysis of Hungarian clade of WNV-2 Central–South–Eastern cluster. In particular, the WNV-2 genomes detected in the Calabria region were characterized by amino acid residues (NS2B-V119I, NS3-H249P, NS4B-S14G, and NS4B-T49A) that were not present in other sequences of the Central–North–Western Europe (including them from NC–Italy). These observed residues were shared with the Central–South–Eastern European WNV reference strain (Hungary 578/10), isolated in 2010 (NS2B-119I, NS3-249P, NS4B-14G, and NS4B-49A). Moreover, among these substitutions, the Proline at NS3-249 was previously associated with higher neuro-virulence.

**Table 1:** Clinical feature of 9 patients with WNV encephalitis admitted to our unit from 2023 to 2025.

Patient (Pt), Sex, Age (Years, y)	Comorbidity	Symptoms	WNV Serology, IgG /IgM	Blood WNV Viremia, Copies/mL	Urinary WNV Viremia, Copies/mL	WNV CSF Viremia, Copies/mL	EEG	Head Tc	Head MRI	Treatment	Complications	Outcome (Days)
Pt 1, F, 71, 2024	HHD, dyslipidemia	Abdominal pain, fever, diarrhea, vomiting, headache	+/+	<500	3028	Neg	NR	UNR	NR	DEX	None	Cured, discharged after 16 d
Pt 2, M, 74, 2024	HHD	High fever, confusion, disorientation, sensory clouding, headache	+/+	Neg	Neg	Neg	Slowing of cerebral bioelectrical activity with additional bursts of spike waves over the left frontotemporal regions	Thin extraaxial hemorrhagic collections along the bilateral fronto-temporo-parietal convexities	Thin bilateral frontoparietal subdural hemorrhagic layers	DEX, Igs	Left hemiparesis, progressively worsening coma	Exitus, 21 days after hospital admission
Pt 3, M, 60,2024	None	High fever, headache, confusion	−/+	67.067	Neg	>50,000,000	NR	UNR	Weak cerebellar leptomeningeal venular enhancement	DEX, Igs	None	Cured, discharged after 21 d
Pt 4, M, 75, 2024	CVE, H	High fever, headache, confusion, dysarthria	+/+	Neg	Neg	Neg	NR	UNR	CVE, cerebral atrophy	DEX, Igs	None	Cured, discharged after 9 d
Pt 5, M, 63, 2023	DM2, CHHD	In the days preceding hospital admission he had suffered; upon arrival at the emergency room he was found to be in a coma (Glasgow Coma Scale 6), with severe weakness of the limbs and high fever	+/+	Neg	Neg	<500	Delta waves spread mainly over the anterior regions EMG: severe sensorimotor polyneuropathy in the 4 limbs, of mixed type, predominantly axonal	UNR	Hyperintensity in the middle cerebellar peduncles, the splenium of the corpus callosum, and the semioval centers bilaterally	DEX, Igs, plasmapheresis	Global clinical worsening with irreversible tetraparesis and mechanical ventilation through tracheostomy	Transferred to a rehabilitation facility 46 days after hospital admission. Exitus after 3 week because of respiratory failure.
Pt 6, M, 84, 2024	Previous right nephrectomy for cancer, prostate cancer undergoing radiotherapy	High fever, headache, confusion	+/+	842.845	9454	Neg	Slowing of cerebral bioelectrical activity	UNR	UNR	DEX, Igs	None	Cured, discharged after 6 d
Pt 7, M, 71, 2025	HHD, cognitive involutional syndrome	High fever, headache, confusion, worsening motor difficulty with lower limb weakness	+/+	Neg	Neg	Neg		ICH of the occipital horns of the lateral ventricles (Figure 2)	Diffuse meningoencephalitis, with involvement of the cauda extremity roots. ICH of the occipitopolar and bihemispheric mid-posterior cingulate sulci, the left temporoparietal carrefour, and the fourth ventricle, occipital horns, and pericerebellar cistern. Posterior dorso-lumbo-sacral, anterior dorso-lumbar (up to L1), and sacral epidural hematoma (Figure 3 and Figure 4)	DEX, Igs	Gradual clinical improvement, however lower limb weakness persisted	Transferred to a rehabilitation facility 34 days after hospital admission.
Pt 8, M, 66, 2025	DM2, CHHD, CVE	High fever, vomiting, peripheral paresis of the VII right cranial nerve	+/+	Neg	Neg	Neg	Mild diffuse encephalic suffering with slowed background electrical activity and bursts of generalized slow activity	CVE	CVE	DEX, Igs	None	Cured, discharged after 20 d
Pt 9, M, 68, 2025	DM2,	High fever, soporific state, poor verbal, tactile and pain reactivity	+/+	Neg	Neg	1072	Slow activity in the theta and delta bands, low voltage, slightly asymmetric (right greater than left).	Bilateral frontal lobe subdural hemorrage, ICH in the right frontotemporal lobe	NR	DEX, Igs	Overall worsening of clinical conditions and comatose state	Exitus, 13 d after hospital admission

Abbreviations: CHHD, chronic hypertensive heart disease; CVE, chronic vasculare encephalopathy; DEX, dexamethasone; DM2, type 2 diabetes mellitus; EEG, elettroencephalophy; EMG, electromyography; H, hypertension; HHD, hypertensive heart disease; Igs, immunoglobulins; Neg, negative; NR, not reported; UNR, uremarkable; WNV, West Nile virus.

**Figure 2:**
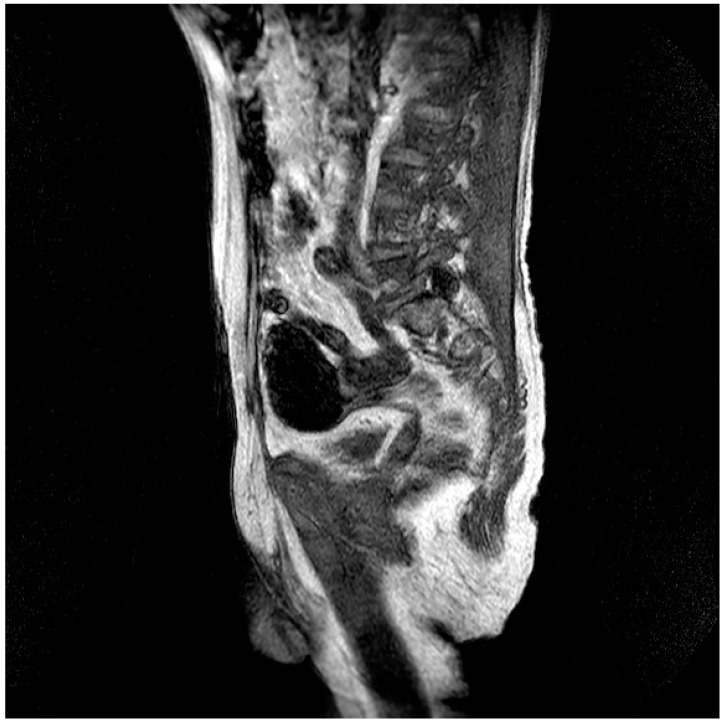
Evidence of an extensive epidural spinal cord hematoma on spinal MRI (Table 1, patient 7).

**Figure 3:**
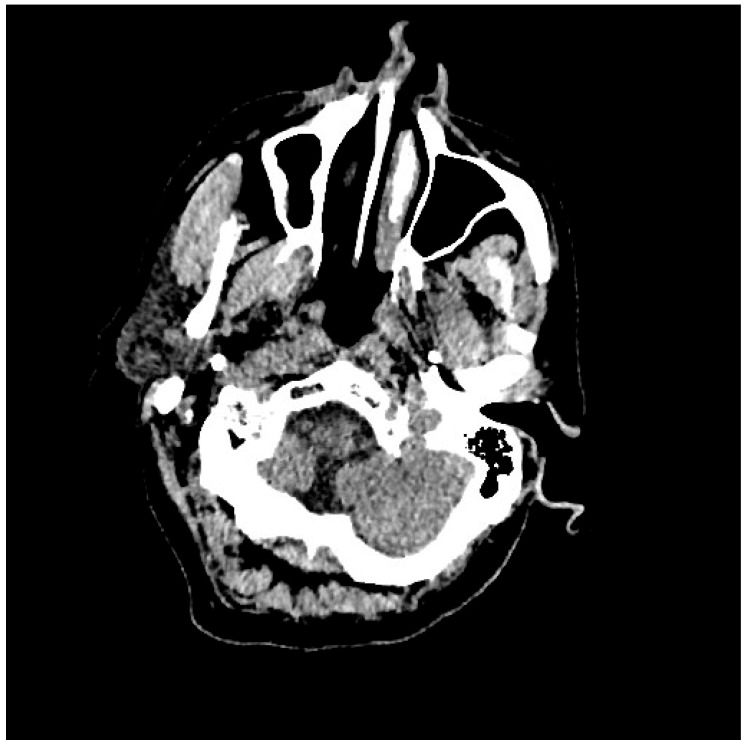
Evidence of ICH on brain CT scan (Table 1, patient 7).

**Figure 4:**
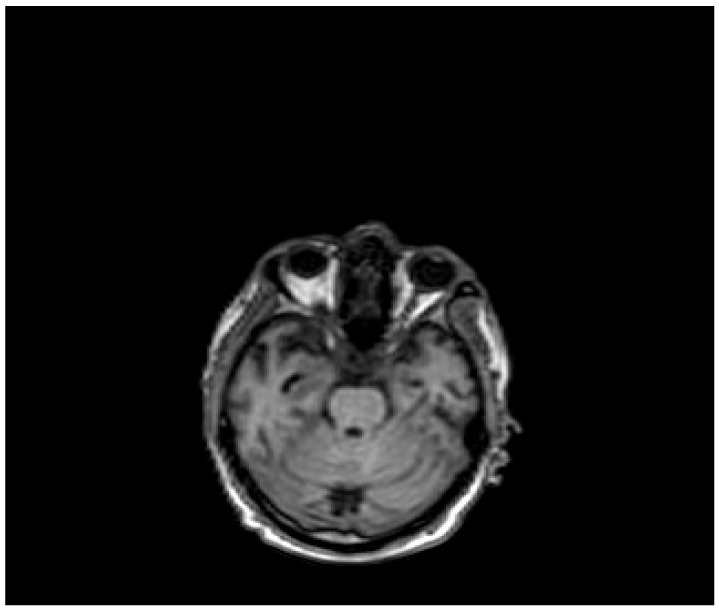
Evidence of ICH on brain MRI (Table 1, patient 7).

## 4. Discussion

A comprehensive search was conducted across the PubMed, Scopus, Web of Science, and Embase databases to extract relevant published data existing about ICH and WNE, up to November 2025. This retrospective analysis reveled only three reported cases of ICH in WNE patients (Table 2).

A case of severe, bilateral necrotizing and hemorrhagic encephalitis was reported in a 43-year-old man, undergoing mycophenolate mofetil therapy for a renal transplant due to diabetes mellitus. He died 12 days after hospital admission. Autopsy revealed predominantly inflammatory involvement of motor neurons and a positive immunohistochemical staining for WNv antigen [4].

Castaldo N et al. [5] documented in 2020 a 57-year-old male patient suffering from autoimmune glomerulopathy, treated with immunosuppressive therapy, with rhombencephalitis and massive intraparenchymal hemorrhage, fourth ventricle compression and tonsillar herniation. He died 5 days after admission [4].

Harroud A. et al. [2] described a 73-year-old female patient with lymphocytic encephalitis and subarachnoid hemorrhage. She was treated with supportive care and he was discharged in improved clinical condition, able to walk without support, but with chronic cognitive deficits.

Our review highlighted nine cases of ischemic and hemorragic stroke associated with WNV infection reported in the literature [6,7,8,9,10,11,12,13] (Table 2).

Seven patients were female and two were male, with a median age of 49.22 years (range, 7–74). The outcome was favorable, being all but one patient discharged, with no residual neurological findings and no recurrences in subsequent follow-up. Only one patient died due to severe acute respiratory failure during the ICU stay. Comorbidities were varied; only one of these patients had a confirmed history of recurrent cerebral ischemic episodes. Tangella N et al. reported a case of acute ischemic stroke and subaracnoid hemorrage (SAH) [12], while Lowe LH et al. [7] described three pediatric cases of primary idiopathic cerebral vasculitis complicated by stroke. These cases were observed in three girls aged 7, 9, and 12 years and were followed by complete recovery with a regimen of aspirin, steroids, and cyclophosphamide. In all three of these children, during follow-up to 36 months, 19 months, and 18 months, respectively, no recurrence of stroke was detected, and the neurological condition was stable [7].

A recent systematic review [14] of case reports on WNV infection-associated cranial nerve (CN) neuropathy reported 13 cases of facial palsy related to WNV infection. Thus, facial palsy represented 31.0% of 42 cases of cranial nerve neuropathies as an outcome of WNV infection, reported in 30 case reports. The mean time until onset of signs and symptoms of facial palsy was 1–16 days [14].

Complete recovery was significantly associated with absence of comorbidities, while it is difficult to establish the role of the use of antivirals, steroids and antibiotics, variously used in combination without a precise criterion. However, it is also true that three male patients aged 57, 45, and 27 who did not receive antivirals did not achieve resolution of facial palsy. Furthermore, a 34-year-old man with involvement of the VII and XI cranial nerves, treated with antibiotics, achieved only partial improvement.

A 63-year-old man developed a GBS unresponsive to steroids, immunoglobulins and plasmapheresis. This patient died after approximately two months after hospital admission because of severe respiratory complications.

WNDD presenting as GBS or Guillain–Barré-like syndrome (GBLS) has been increasingly reported [15,16,17,18,19,20,21,22,23,24] (Table 3 and Table 4).

A polio-like syndrome with paralysis involving one (monoparesis) to four limbs (tetraparesis), with or without brainstem involvement and respiratory failure, has been described. This syndrome of acute flaccid paralysis may occur without overt fever or meningoencephalitis. Although involvement of the anterior horn cells of the spinal cord and motor neurons of the brainstem are the main sites of pathology responsible for neuromuscular signs, inflammation may also involve the skeletal and/or cardiac musculoskeletal system (myositis, myocarditis), motor axons (polyradiculitis), and peripheral nerves (GBS), or present as brachial plexopathy. Furthermore, involvement of spinal sympathetic neurons and ganglia provides an explanation for the autonomic instability observed in some patients. Many patients also experience prolonged subjective generalized weakness and disabling fatigue. The long-term outcome of WNE polio-like syndrome appears to be more heterogeneous than preliminary data may have suggested, with some patients showing little neurologic and functional improvement and others showing substantial improvement. The degree of initial weakness appears to be a predictor of subsequent long-term outcome. The Centers for Disease Control and Prevention (CDCs) report that 13% of WNV infections manifest as GBS [25].

In most of the cases reported from the current literature through a PUBMED/MEDLINE search summarized in Table 4, gradual and slow improvement occurred, often with residual neurologic deficits of varying severity. Treatment includes supportive care and consideration of the use of intravenous immunoglobulin and, if unsuccessful, plasmapheresis [15,16,17,18,19,20,21,22,23,24,25].

**Table 2:** Neuroinvasive West Nile virus infections presenting as ischemic or hemorragic stroke.

References, Country, Type of Article	Age (y), Sex	Comorbidity	Clinical Features	Head CT	EMG	Head MRI	Clinical Diagnosis	Autopsy Findings	Treatment	Outcome
Smith R.D. et al. [4], University of Cincinnati, USA, 2024	43, M	Renal allograft, end-stage renal disease from diabetes mellitus	A 2-day history of nausea, vomiting, diarrhea, and chills with fever	Bilateral thalamic edema extending to the midbrain and pons	NR	Extensive edema involving the pons, medulla, midbrain, and bilateral thalami as well as the medial left temporal lobe		A severe, bilateral, necrotizing and hemorrhagic encephalitis preferentiallving motor neurons. Immunohistochemistry search for WNV antigen was positive	Acyclovir therapy, and discontinuation of the immunosuppressive regimen (Micophenolate)	Died 12 days after hospital admission.
Whitney E.A. et al. [8], Emory University, Atlanta, USA, 2006	68, F	Recurrent transient ischemic attacks, peripheral vascular disease, seizures	High fever, cough, losing of balance and falling easily when walking	Non-revealing	NR	Unremarkable	Community-acquired pneumonia, atrial fibrillation, and cerebellar stroke		Oral antibiotics, carbamazepine.	Discharged on the 7th day
Peters S. & Brown K. [11], University of Calgary, Canada, 2021	57, M	None	Pharyngitis and a descending maculopapular rash on the torso, arms, legs, and feet including the palms. Right hemiplegia, aphasia.	“T” occlusion of the distal left internal carotid artery	NR	Patchy infarction in the left insula, basal ganglia, and operculum	Acute cryptogenic stroke		Intravenous thrombolysis, endovascular thrombectomy, intra-arterial verapamil	Discharged with no residual neurologic deficits and no recurrence after two years later
Kulstad E.B., Wichter M.D. [6], Advocate Christ Medical Center, Oak Lawn, Illinois, USA, 2003	70, M	Chronic lymphocytic leukemia	Mental confusion, dysarthria, pronation of the right upper limb, external rotation of the right lower limb, ascending Babinski reflex	Mild atrophy consistent with the patient’s age, some mild chronic ischemic changes in the periventricular white matter	NR	Mild chronic ischemic demyelination with several small lacunar infarcts, but no acute changes	Stroke with rhabdomyolysis and acute renal failure		Oxygen, fluid hydration, intubation.	Death on hospital day 10 because of respiratory failure in ICU
Alexander J.J. et al. [9], University of Missouri, Kansas City, USA, 2006	9, F	Environmental exposure to mosquitoes	Intermittent right arm and leg weakness. She fell from her bicycle and developed transient aphasia	A small hypodense area in the left anterior temporal lobe	NR	Increased T 2- weighted signal in the left caudate nucleus, lentiform nucleus, and left anterior temporal region. Bilateral irregularities of the distal middle cerebral arteries, left posterior cerebral artery, and left middle cerebral artery	Stroke Associated With Central Nervous System Vasculitis After West Nile Virus Infection		She was initially treated with hydration, low-dose aspirin, and verapamil. Methylpredni- solone was started on the 3rd day for probable vasculitis. Five monthly doses of cyclophosphamide began with a moderate improvement in right motor function	Discharged. Clinical improvement 18 months later. Mild left brain volume loss, persistent middle cerebral artery asymmetry, a small left M1 mainstem trunk, and attenuated distal sylvian branches were present in the follow-up
Castaldo N. et al. [5] Udine University, Italy, 2020	57, M	Autoimmune glomerulonephritis in immunosuppressive treatment	Fever, confusion, diplopia, opsoclonus, multifocal myoclonus and generalized tremor	Massive intraparenchymal hemorrhage, fourth ventricle compression and tonsillar herniation	Slow bilateral diffuse slow waves	Unremarkable	Rhombencephalitis, coma, intracranial hemorrhage	Macroscopic examination of the brain showed diffuse malacia	Empirical therapy with ampicillin, ceftriaxone, acyclovir and dexamethasone. Therefore, IVIGs and steroids	Died 5 days after admission
Jacob S. et al. [10], Mayo Clinic, Phoenix, Arizona, USA, 2019	67, F	None. Significant history of pigeon exposure	Right-sided facial droop, right-sided weakness, low back pain, fever and lethargy	Unremarkable	N.A.	Medial left frontal acute infarct	Stroke with encephalopathy		IVIG	Her mental status significantly improved and she was discharged to a rehabilitation facility
Harroud A. et al. [2], Montreal Neurological Hospital and McGill University, Montreal, Canada, 2019	73, F	A remote history of renal cell and breast carcinomas, both in complete remission and no treatment	Confusion, high fever; decreased level of consciousness and aspiration pneumonia requiring intubation. On day 8, the patient developed generalized myoclonus	Unremarkable	Severe slowing but no epileptic activity	Extensive and confluent leukoencephalopathy and interval appearance of bilateral convexity. SAH	Encephalitis with lymphocytic pleocytosis and myoclonus		Supportive treatment including neurointensive care monitoring and IV hydration	On discharge, the patient was able to walk without support but suffered from residual cognitive deficits
Hingorani K. et al. [13], Boston Medical Center, Massachusetts, USA, 2023	70, F	None	Depressed level of consciousness, hypophonia	Bilateral corona radiata strokes	Mild generalized delta slowing	Bilateral corona radiata strokes	Stroke		NR	Discharged
Tangella N et al. [12], Rutgers The State University of New Jersey, USA, 2023	74, M	ESRD, T2DM, DDRT, prostate cancer	3–4 days of nausea, vomiting, diarrhea, fever and chills	NR	Diffuse slowing	Acute ischemic stroke and SAH	NR		Empiric meningitis treatment. Therefore, IVIG for suspected GBS	NR
Lowe L.H. et al. [7], University of Missouri–Kansas City, USA, 2005	7, F	None	Headache, right hemiparesis, aphasia, and facial droop	Unremarkable	NR	Acute left middle cerebral artery stroke	Primary cerebral vasculitis		Aspirin, steroids, cyclophosphamide	Discharged, without recurrent stroke after 36 months of clinical follow-up
Lowe L.H. et al. [7], University of Missouri–Kansas City, USA, 2005	12, F	None	Headache, slurred speech, nausea, and vomiting	Abnormality in the left middle cerebral artery, internal carotid artery, and anterior carotid artery distributions	NR	Abnormality in the left middle cerebral artery, internal carotid artery, and anterior carotid artery distributions	Primary cerebral vasculitis		Aspirin, steroids, and cyclophosphamide	Discharged, without recurrent stroke after 18 months of clinical follow-up
Lowe L.H. et al. [7], Missouri–Kansas City, USA, 2005	9, F	None	Headache, right arm and right leg weakness, and acute aphasia	Acute left middle cerebral artery distribution stroke	NR	Acute left middle cerebral artery distribution stroke	Primary cerebral vasculitis		Aspirin, steroids, and cyclophosphamide	Discharged, without recurrent stroke after 19 months of follow-up

Abbreviations: CT, computerized tomography; DDRT, deceased donor renal transplantation; EMG, electromyography; ESRD, end-stage renal disease; IVIG, intravenous immunoglobulin therapy; y, year; MRI, magnetic resonance imaging; NR, not reported; SAH, subarachnoid hemorrhage; T2DM, diabetes mellitu type 2; WNV, West Nile virus.

**Table 3:** Neuroinvasive West Nile virus presenting as Guillain–Barré syndrome (GBS) or Guillain-Barré-like syndrome (GBLS).

References, Country, Type of Article	Age (y), Sex	Comorbidity	Clinical Features	CT Scan of the Brain	EMG	MRI of the Brain/Spine	Treatment	Outcome
Ashkin A. et al., 2023 [17], USA, case report	67, M	CAD, hyperlipidemia	Fever, nausea, vomiting, and right lower quadrant abdominal pain	NA	Nonrecordable nerve conduction velocity in bilateral peroneal nerve, a slowing of the right tibial nerve conduction velocity	Not remarkable	3-day course of IVIG, 1 g of methylprednisolone daily for of 5 days	Residual lower extremity weakness
Sciturro M. et al., 2022 [18], Florida, USA, case report	64, M	Asthma, diverticulitis, nephrolithiasis	Generalized bilateral upper and lower extremity weaknes	NA	NA	Not remarkable	IVIG and plasmapheresis, with no improvement	Died
Beshai R. et al., 2020 [19], New York, USA, case report	65, F	NA	Progressive ascending paralysis	Normal	Acute sensorimotor axonal and demyelinating peripheral neuropathy	NA	10-day course of IVIG	Improved, but lower extremity weakness unchanged
Paphitou N.I. et al. [23] 2017, Cyprus, case report	75, M	CAD, prostate cancer	Reduced muscle strength in the lower limbs	Not remarkable	Nonspecific findings of peripheral neuropathy	Not remarkable	5-day course of IVIG	Recovered
Walid M.S. et al., 2009 [24], USA, case report	55, M	Diabetes mellitus, hypothyroidism	Muscle weakness and numbness in all four extremities	Not remarkable	Sensorimotor mixed polyneuropa- thy, predominantly axonal	Not remarkable	Plasma- pheresis and dexamethasone, with no improvement. A 7-day course of IVIG with improvement	Recovered
Sejvar J.J. et al., 2006 [15], Colorado, USA, prospective study	4 pts	NA	Ascending weakness with sensory symptoms	NA	Demyelinating sensorimotor neuropathy	NA	NA	1 pt lost to follow-up. 2 pts had recovery
Ahmed S. et al., 2000 [20], USA, case report	69, M	Hypertension	Progressive weakness, quadriparesis	Not remarkable	Demyelinating polyneuritis with secondary motor axon degeneration	Not remarkable	5 cycles of plasmapheresis with no improvement; 2 courses of IVIG, with only minimal improvement	Transferred to a nursing home with a tracheostomy and a gastrostomy feeding tube
Joseph N. et al., 2019 [21], USA, case report	40, M	Hypertension	Progressive muscle weaknes	NA	Demyelinating sensorimotor polyneuropathy	NA	5-day course of IVIG	Recovered
Abraham A. et al., 2011 [22], USA, case report	67, F	None	Shoulder and back pain, generalized weakness, fever and diarrhea	Occipital lobes hypodensities	Demyelinating polyneuropathy	PRES	5-day course of IVIG	Recovered

Abbreviations: CAD, coronary artery disease; CT, computerized tomography; EMG, electromyography; IVIG, intravenous immunoglobulin therapy; y, year; MRI, magnetic resonance imaging; NA, not available; PRES, posterior reversible encephalopathy; pts, patients.

**Table 4:** Summary of cases of facial palsy associated with WNV infection, January 2000 to November 2025.

References (First Author, Year, Country)	Age (Years, y), Sex	Medical History	Clinical Features	Cranial Nerves	Treatment	Outcome
Flaherty M.S. et al., 2003 [26], USA	34, M	CD	Non-specific viral illness, tinnitus, facial palsy	VII, XI	Systemic antibiotics	Partial recovery
Rosenheck M.L. et al., 2022 [27], USA	40, F	CD	Facial palsy, weakness in extremities	VII	Systemic antivirals, steroids and antibiotics	Cured
EL-Dokla A.M. et al., 2018 [28], USA	48, M	NA	Facial palsy, weakness in extremities	VII	Systemic antivirals snd steroids	Cured
EL-Dokla A.M. et al., 2018 [28], USA	49, F	NA	Non-specific viral illness, facial palsy weakness in extremities	VII	Systemic antivirals and steroids	Cured
Sejvar J.J. et al., 2003 [29], USA	57, M	CD	Facial palsy weakness in extremities	VII	Supportive therapy	No recovery
Li J. et al., 2003 [30], USA	45, M	Healthy	Facial palsy	VII	Immunoglobulins	No recovery
Li J. et al., 2003 [30], USA	27, M	CD	Non-specific viral illness, facial palsy, weakness in extremities	VII, XI	NA	No recovery
Al-Hashimi I. et al., 2024 [31], USA	68, F	CD	Non-specific viral illness, facial palsy, diplopia, decreased shoulder shrug, dysarthria	II, VII, XI	Systemic antivirals and antibiotics	Cured
Cunha B.A. et al., 2006 [32], USA	47, M	NA	Vision problems	VI, VII	NA	Cured
Nikolic N. et al., 2024 [33], Serbia	65, F	CD	Non-specific viral illness, facial palsy, weakness in extremities	VII	Systemic antivirals and antibiotics	Cured
Ostapchuk Y.O. et al., 2020 [34], Kazakhstan	28, M	NA	Non-specific viral illness, facial palsy weakness in extremities	VII	Supportive therapy	Cured
Ostapchuk Y.O. et al., 2020 [34], Kazakhstan	19, F	NA	Non-specific viral illness, facial palsy, weakness in extremities	VII	Systemic antivirals and supportive therapy	Cured
Jhunjhunwala K. et al., 2018 [35], USA	28, F	Healthy	Non-specific viral illness, facial palsy, weakness in extremities	VII	Systemic antibiotics	Cured
This case series	66, M	CD	High fever, vomiting, peripheral paresis of the VII right cranial nerve	VII	Steroids, Igs	Cured

Abbreviations: CD, chronic disease; F, female; M, male; NA, not available.

## 5. Conclusions

Preliminary data do not allow us to determine whether our strains presented an increased neurovirulence, potentially attributable to some amino acid substitutions located in the Envelope protein. However, additional investigations and data consolidation are necessary to clarify this point. Most WNV infections are asymptomatic, mild, or undiagnosed; however, severe cases of WNDD are increasingly reported, probably related to WNV’s specific affinity for the nervous system, both central and peripheral. WNV infection can rarely cause cerebral vasculitis, a serious inflammation of brain blood vessels, leading to complications like ischemic or hemorrhagic stroke and severe neurological issues [2]. This viral vasculitis may involve direct viral infection of endothelial cells, causing vessel damage, rupture, and subsequent bleeding or blockage. Clinicians should suspect WNE in ischemic and hemorragic stroke cases during summer and mosquito season, especially with cerebral hemorrhagic signs. We suggest that clinicians should be vigilant for vasculitic complications in WNE patients, including ICH.

## Data Availability

For ethical and privacy considerations, and for the purposes of remaining in accordance with the approval provided by the institutional ethics committee, these data cannot be publicly shared. The restriction is necessary because the raw qualitative data contain sensitive and potentially identifiable information about the study participants.

## Abbreviations

The following abbreviations are used in this manuscript:

CAD	coronary artery disease
CD	chronic disease
CHHD	chronic hypertensive heart disease
CNs	cranial nerves
CSF	cerebrospinal fluid
CT	computerized tomography
CVE	chronic vasculare encephalopathy
DEX	dexa-methasone
DHD	dilated heart disease
DM2	type 2 diabetes mellitus
EEG	elettroen-cephalophy
EMG	electromyography
F	female
GBS	Guillain–Barré syndrome
H	hypertension
HHD	hypertensive heart disease
ICH	intraparenchimal cerebral hemorrhage
Igs	immunoglobulins
IVIG	intravenous immunoglobulin therapy
M	male
MRI	magnetic resonance imaging
NA	not available
Neg	negative
NR	not reported
PRES	posterior reversible encephalopathy
PTS	patients
UNR	uremarkable
WNND	West Nile neuroinvasive disease
WNE	West Nile encephalitis
WNV	West Nile virus
Y	year
